# Psilosis or Sprue

**Published:** 1899-09-16

**Authors:** 


### PSILOSIS OR SPRUE.

Among the discussions upon tropical diseases at the Portsmouth meeting of the British Medical Association a leading place must be given to that upon psilosis or sprue, which was opened by an exhaustive paper on the subject by Dr. George Thin, president of the section. He maintained that the disease was not met with in temperate climates, for although he had been told by distinguished physicians that they had observed cases in persons who had never been out of England, he had not been able to find in medical literature dealing with diseases occurring in this country any account of cases answering to psilosis as we know it. Dr. Thin divided cases of psilosis into two categories, one type being more common in the Eastern Archipelago, and the other being more commonly met with in India. In the former the symptoms connected with the mouth, the tongue, and the throat were those which were most early prominent, and even the most pronounced and disagreeable; while in the other copious watery stools were the chief feature, the mouth and tongue symptoms not manifesting themselves in a decided form until a later stage of the disease, cases, indeed, being sometimes met with in which, even after years, the tongue and mouth might be still free from specific inflammation. Thus in the class of cases which might be called psilosis linguæ, discomfort in swallowing, felt in the mouth, throat, and gullet, was often very great long before the debility or emaciation was marked; while in the Indian type, which took the form of diarrhœa or white flux, the mouth symptoms were usually synchronous with advanced malnutrition and emaciation. It was, however, to be noted that there was a form of tropical diarrhœa which came on gradually in elderly persons, or in those who had been long resident in the tropics, which was distinct from psilosis, although in it also, when the characteristic looseness of the bowels had lasted for a considerable time, a gradual thinning of the epithelial covering of the tongue with considerable rawness might tape place. Again, it should be mentioned that psilosis might occur for the first time in people who had previously been in the East long after they had returned to this country. Such cases were apt to be met with in elderly people when the powers begin to fail. As to the etiology of the disease this was unknown. It was clearly not due to the age of the patient nor to the mere fact of living in high temperatures. The disease was associated with residence in certain parts of the world, and was absent in other parts. One of the effects of its specific poison was to alter the secretion in the small intestine and to allow the food bolus to retain its acidity. The activity of the poison might be greatly increased by farinaceous and animal foods, but it might be starved out by limiting the patient's diet, and it appeared not to grow if the food consisted exclusively of milk.

Dr. W. J. Buchanan dealt with the disease as it occurs among the natives in India, among whom he thought it was very common, and often ran a rapid course. He thought that the condition known as "famine diarrhœa" was essentially the same in its symptoms and ultimate results as sprue, and also that in many cases of relapsing dysentery a condition strongly resembling fully-developed psilosis was met with, the frothy pultaceous diarrhœa characteristic of sprue alternating with the dysentery. He thought that many cases, if caught in time, were cured; and that although the treatment traditional in Bengal gaols, namely milk alone, was necessary for natives who would not touch meat, the all-meat-juice diet lately recommended by Dr. Cantlie was preferable for Europeans.

Dr. Cantlie said that for several years past he had discarded milk, for although it relieved the symptoms it did not cure the disease, and its prolonged use was calculated to cause the latter to continue indefinitely. At whatever stage patients suffering from sprue came under his care he immediately put them on a meat diet. If the patient were very ill and confined to bed he had him fed every quarter or half hour with raw meat juice, scraped beef, freshly-made beef essence, raw meat wafer sandwiches, and some plain jelly. But as the weakness subsided, or if the patient were not so ill, the intervals between the feedings might be longer and the quantity greater, and as soon as possible finely-minced beef, rapidly heated over a quick fire, was to be substituted, calf's-foot jelly or plain jelly being allowed *ad libitum* between meals and in the night. A gradual restoration to ordinary diet was thus effected, and it was found that, in place of the white stools which under a milk diet continued for long to be scarcely fæcal in character, the meat diet immediately brought down a dark coloured bilious motion, thus showing that the digestive organs were being gradually brought into play and their functional activity re-established.

In reply Dr. Thin insisted upon the advantages of milk in the more active phases of the disease. There was a time when meat was well borne, but that was, he said, when the specific condition no longer existed, and only the sequelæ remained.

